# The breakup of a long-period comet is not a likely match to the Chicxulub impactor

**DOI:** 10.1038/s41598-022-12873-3

**Published:** 2022-06-21

**Authors:** Steven J. Desch, Alan P. Jackson, Jessica L. Noviello, Ariel D. Anbar

**Affiliations:** 1 School of Earth and Space Exploration, Arizona State University, Tempe, AZ 85287-1404 USA; 2 NASA Postdoctoral Management Fellow, Goddard Space Flight Center, Greenbelt, MD 20771 USA; 3 School of Molecular Sciences, Arizona State University, Tempe, AZ 85287-1604 USA

**Keywords:** Meteoritics, Asteroids, comets and Kuiper belt, Natural hazards

**arising from**: A. Siraj and A. Loeb; *Scientific Reports* https://doi.org/10.1038/s41598-021-82320-2 (2021).

## Introduction

Since the discovery of Ir in the clay layer at the K-Pg boundary^1^, scientists have sought to constrain the origin of the extraterrestrial impactor that triggered the end-Cretaceous mass extinction of the non-avian dinosaurs and other species. While the first proposal was for an asteroid^1^, for a while some theories invoked a cometary impactor to explain perceived periodicities in mass extinctions^2^. Such models have long been disfavored by the mass of Ir in the layer, inferred to be $2.0{-}2.8 \times 10^{11} \, \mathrm{g}$^3^. The size of the Chicxulub crater leads to an estimated asteroid impactor diameter, $D \approx 10 \, \mathrm{km}$^1,4,5^. Comets typically impact at higher speeds, reducing the impactor mass for the same impact energy^4^. Although it is increasingly recognized that a continuum exists between comets and asteroids, ’comets’ are considered to be more ice-rich (estimates for 67P/Churyumov-Gerasimenko are about 20%;^6^), implying lower Ir contents per impactor mass. A carbonaceous chondrite-like asteroid of the appropriate size would likely deliver $\approx 2.3 \times 10^{11} \, \mathrm{g}$ of Ir^4^, in the center of the estimated mass range of the global Ir layer; but a comet would only deliver $\sim 0.1 \times 10^{11} \, \mathrm{g}$, because it would be less massive. Although these conclusions are long standing, Siraj and Loeb^7^ have recently argued anew in favor of a comet over an asteroid, based on dynamical and geochemical evidence. Here we demonstrate that their arguments are based on misinterpretations of the literature, and that an asteroid is in fact still highly favored over a comet.

## Geochemical arguments

Siraj and Loeb^7^ cite good evidence that the Chicxulub impactor was carbonaceous chondrite-like, but then assert that 100% of comets satisfy this constraint but only 10% of asteroids do. This assertion conflates carbonaceous chondrites with specific types (CB, CH, CI, CM, CO, CR, CV) of carbonaceous chondrites. It underestimates the fraction of asteroids that match the Chicxulub impactor’s composition, and/or overestimates the fraction of comets that would.

Siraj and Loeb^7^, citing Bottke et al.^8^, claim only 30% of asteroids are C-type (spectrally resembling carbonaceous chondrites) and appear to imply that only 40% of carbonaceous chondrites are the specific type CM associated with the impactor. In fact the fraction of near-Earth asteroids that are C-type is ${\sim 40{-}50\%}$^9^. As well, the Chicxulub impactor could be CM- *or* CR-like. Siraj and Loeb^7^ cite evidence from a fossil meteorite in the K-Pg clay layer, which demands the impactor be CV, CO, CR, or possibly CM, but not CI^10^. They also cite evidence from the $\epsilon ^{54}\mathrm{Cr}$ isotopic anomaly in the K-Pg clay clayer, which argues the impactor was CM (and CR, CH, and CB have the same $\epsilon ^{54}\mathrm{Cr}$), but argues against CV, CO, and CI^11^. The authors could have cited equally strong arguments from platinum-group element patterns, which favor CM or CO (and allow CR), but rule out CI^12^. The composition of the Chicxulub impactor is a match to either CM or CR chondrites. Siraj and Loeb^7^ argue that CM chondrites comprise a fraction $\approx 40\%$ of all carbonaceous chondrites, based on statistics of intact falls; but a larger fraction of C-type asteroids may match CM or CR chondrites. At a minimum, ${\approx 40{-}50\%}$ of near-Earth asteroids are carbonaceous chondrite-like, and $>20\%$ of asteroids striking Earth match the specific composition of the Chicxulub impactor.

Siraj and Loeb^7^ claim 100% of comets are carbonaceous chondrite-like, which may be loosely true; but comets are not definitively associated with any particular subtype of carbonaceous chondrite. They are most strongly associated with carbonaceous chondrites of type CI, based on their low albedo, friability, lack of chondrules, presence of anhydrous silicates, and low impact rate on Earth^13^. A comet-like origin has been argued for CI chondrites like Orgueil^14^, and indeed the reflectance spectrum of the refractory crust of 67P/Churyumov-Gerasimenko is most similar to the insoluble organic material of Orgueil^15^. There nevertheless remain significant differences between 67P and CI chondrites and carbonaceous chondrites in general^15^, and comets do not need to conform to any carbonaceous chondrite; but of them, they most closely resemble CI chondrites. Notably, none of the lines of geochemical evidence above is consistent with CI chondrites, indicating that while 100% of comets may be carbonaceous chondrite-like, possibly 0% of them match the specific composition of the Chicxulub impactor in detail.

The net effect is that Siraj and Loeb^7^ applied differing standards to the geochemical evidence for asteroids and comets. If the impactor must simply be carbonaceous chondrite-like, then comets are more likely (for a given impact rate) by a factor of 2, not 10. If the impactor must specifically match a CM or CR composition, then $> 20\%$ of asteroids provide a match, but perhaps no comets do.

Crucially, the mass of Ir in the clay layer likewise is a match to an asteroidal impactor, but not a comet.

## Dynamical arguments

Siraj and Loeb^7^ downplay the frequency with which asteroids impact Earth, and overestimate the likelihood of a comet impact. The authors state that the Chicxulub impact was the single largest impact in the last 250 Myr, and that asteroids with $D= 10$ km should impact the Earth with mean rate once per $\sim 350$ Myr. Therefore by their own numbers the probability of a $D > 10$ km asteroid impacting Earth in the last 250 Myr is $> 50\%$. Whatever the probability of a comet impact, an asteroid impactor is a probable event.

The main point of Siraj and Loeb^7^ is that a significant fraction, $\sim 20\%$, of long-period comets (LPCs) impacting the Earth will have first passed through the Sun’s Roche limit and fragmented into a number, *N*, of smaller comets, potentially increasing the probability one will strike Earth. A comet *N* times more massive than the final Chicxulub impactor is rarer than an undisrupted LPC with the size of the Chicxulub impactor, by a factor of $(N^{1/3})^{1-q}$, where $q \approx 2.0{-}2.7$; but because there are more fragments, this would increase the rate of Chicxulub-scale impactors by a factor $\approx 0.2 \, \times \, N \, \times \, N^{(1-q)/3}$, which is $\approx 15$ for $q = 2$ and $N = 630$ (equivalent to a 60 km-diameter comet breaking up into ones with diameter 7 km). The authors state that undisrupted LPCs the size of the Chicxulub impactor ($D = 7$ km) are expected to impact Earth once every 3.8–11 Gyr, so only if $N \sim 10^3$, enhancing the fluxes by factors $> 15$, is the collision timescale < 250 Myr and comparable to asteroids.

Despite its central importance, the choice of $N \approx 630$ appears unjustified. The analytical treatment of Hahn & Rettig^16^ shows the number of fragments generated is fixed during the encounter, by the relative timescales of spreading and gravitational contraction, which are functions of the comet’s density, $\rho _0$, and its perihelion distance, $r_0$. The contraction timescale, $t_\mathrm{contr}$, in units of the encounter timescale, $\tau =(G \rho _\mathrm{c})^{-1/2}$, is $t_\mathrm{contr} / \tau \approx 0.94 \, (\rho _\mathrm{c}/ \rho _0)^{1/2} \, N^{1/2}$, where $\rho _\mathrm{c} = (1 M_{\odot }) / r_0^3$. The spreading timescale in units of the encounter timescale is found by numerical simulation and appears to be $t_\mathrm{spread} / \tau \approx 0.7 \, N^{0.85}$, assuming the dimensionless treatment applies to the Sun as well as Jupiter. A disrupted comet coalesces into fragments when these timescales are equal, which is when $N \approx 2.3 (\rho _\mathrm{c} / \rho _0)^{1.43}$. The closer to the Sun the comet penetrates, the more fragments are produced, but the minimum value of $r_0$, $1 R_{\odot }$, corresponds to $\rho _\mathrm{c} = 5.9 \, \mathrm{g} \, \mathrm{cm}^{-3}$. Assuming the authors’ $\rho _0 = 0.7 \, \mathrm{g} \, \mathrm{cm}^{-3}$, the *maximum* number of fragments that can be produced by tidal disruption is $\sim 50$, for comets unrealistically skimming the Sun’s photosphere. Assuming a more typical $r_0 \approx 0.7 \times$ the Roche limit, $N \approx 12$ is more likely. That this is similar to the number of fragments produced in the tidal disruption of Shoemaker-Levy 9 is unsurprising since Jupiter and the Sun are of similar density. Note that^17^, cited by Siraj and Loeb^7^ for cometary density, actually give a range of 0.5–0.7 g cm$^{-3}$ and data from the *Rosetta* spacecraft has shown 67P/Churyumov-Gerasimenko to have a density of 0.538 g cm$^{-3}$^18^. The estimate $N \sim 10^{3}$ made by Siraj and Loeb^7^ appears to be based on a misinterpretation of Hahn and Rettig^16^, somehow **incorrectly** and **mistakenly** setting *N* equal to $t / \tau$, where *t* is the time for the fragments to reach Earth.

In addition, applying the formalism of Hahn and Rettig^16^ to the case of a $D = 60$ km comet rounding the Sun, the length of the debris chain once it reaches Earth would be roughly 50 Earth diameters. Earth would have to intercept roughly 1/50 of the fragments, so if there were 630 fragments in the debris chain, Earth would intercept roughly 13 of them. This would demand that the Chicxulub impact be one of a chain of $\sim 13$ equally large craters on Earth, which is not observed.

Considering observed instances of tidal disruption, the tidal disruption of comet Shoemaker-Levy 9 by Jupiter produced roughly 20 fragments. Similarly, 13 crater chains on Ganymede and Callisto have been interpreted as resulting from impacts by tidally disrupted comets, with each consisting of between 6 and 25 craters (with a mean of 11)^19^. Perhaps the most directly relevant to the case at hand are the sungrazing comets. The Kreutz family of sungrazing comets has 9 known large members, believed to have been created through a succession of disruption events over the last few thousand years^20,21^. There are many more small (<100 m diameter) members of the Kreutz family, but these account for a small fraction of the total mass^22^. All of the available evidence suggests that tidal disruption events produce a small number, nowhere near 630, of large fragments.

## Summary

Siraj and Loeb^7^ make a valid point that a Chicxulub-scale cometary impactor ($D = 7$ km) may be not *quite* as uncommon as previously thought, because some fraction of comets may be tidally disrupted by passage within the Sun’s Roche limit. Similar ideas were expressed 30 years ago by Bailey et al.^23^. But even setting $q=2$ and $r_0 = 1 R_{\odot }$, so that $N=50$, the enhancement in flux is only a factor $< 4$; and using the more likely $N=12$, the enhancement is only a factor of 2. The mean timescale for an impact with a Chicxulub-scale comet is most likely $> 2$ Gyr, while the mean timescale with an asteroid remains $\sim 350$ Myr.

Siraj and Loeb^7^ effectively applied different standards to the geochemical evidence for comets and asteroids. If only a loose match to a carbonaceous chondrite is demanded, then comets are only a factor of 2, not 10, more likely than asteroids (for the same impact rate). If it is demanded that the impactors match a CM or CR carbonaceous chondrite composition, then $>20\%$ of asteroids, but possibly $\sim 0\%$ of comets, are a match. As well, Siraj and Loeb^7^ cite Alvarez et al.^1^ but do not even discuss the evidence from the iridium in the K-Pg clay layer that is the point of that paper, which favors an asteroidal impactor but strongly disfavors a comet, which only supplies about 4% as much iridium as an asteroid^4^.

There is a $>50\%$ probability a $D=10$ km asteroid would have hit the Earth in the last 250 Myr. Among Earth-crossing asteroids, ${\approx 40{-}50\%}$ are C-type, associated with carbonaceous chondrites. At least $40\%$ of C-type asteroids, possibly more, will be of the type CM or CR that match the Chicxulub impactor. In contrast, even after including tidal disruption, the mean timescale for impacts by $D = 7$ km comets is $> 2$ Gyr, in tension with the recency of the Chicxulub impact, as there is only a $\sim 10\%$ probability of such an impact in the last 250 Myr. Because of the flaws in their interpretation of the literature, the dynamical and geochemical arguments presented by Siraj and Loeb^7^ do not change the consensus that an asteroid, not a comet, struck the Earth 66 Myr ago.
